# *Mycobacterium microti* Infection in Free-Ranging Wild Boar, Spain, 2017–2019 

**DOI:** 10.3201/eid2511.190746

**Published:** 2019-11

**Authors:** Bernat Pérez de Val, Albert Sanz, Mercè Soler, Alberto Allepuz, Lorraine Michelet, María Laura Boschiroli, Enric Vidal

**Affiliations:** Institut de Recerca i Tecnologia Agroalimentàries–Centre de Recerca en Sanitat Animal, Bellaterra, Spain (B. Pérez de Val, A. Allepuz, E. Vidal);; Departament d’Agricultura, Ramaderia, Pesca i Alimentació de la Generalitat de Catalunya, Barcelona, Spain (A. Sanz, M. Soler);; Universitat Autònoma de Barcelona, Bellaterra (A. Allepuz);; Agence Nationale de Sécurité Sanitaire de l’Alimentation, de l’Environnement et du Travail, Maisons-Alfort, France (L. Michelet, M.L. Boschiroli)

**Keywords:** Bacteria, France, *Mycobacterium microti*, Spain, Tuberculosis and other mycobacteria, Wild boar

## Abstract

*Mycobacterium microti* is a member of the *Mycobacterium tuberculosis* complex that causes pathology in many mammals. *M. microti* infections have been found in some countries in Europe. We report an outbreak of tuberculosis caused by *M. microti* in wild boars in Spain.

*Mycobacterium microti* is a member of the *Mycobacterium tuberculosis* complex (MTBC), which also includes *M. tuberculosis* and *M. bovis*, the main causes of human and animal tuberculosis (TB), respectively. Even though voles and other wild small rodents were initially identified as its natural hosts (*1*), *M. microti* can cause pathology in a wide range of mammals, including pets, livestock, wildlife (*2**–**5*), and humans (*6*). *M. microti* infections have been previously reported in several countries in Europe, including Switzerland, Italy, and France (*3*,*7**–**9*). We report an outbreak of tuberculosis caused by *M. microti* in free-ranging wild boars in the Iberian Peninsula in Spain. 

During June 2017–March 2019, a total of 9 free-ranging wild boars with lesions associated with TB were detected in the outbreak area, covering ≈3,000 hectares in the Catalan Pyrenees (Figure). TB was confirmed histologically, first by hematoxylin and eosin staining (9/9) and then by Ziehl-Neelsen staining of acid-fast bacilli (7/9). In all cases, submandibular lymph nodes showed granulomatous necrotizing lymphadenitis, sometimes with scant acid-fast bacilli, similar to that found in *M. microti* infections previously described in wild boar, which were generally confined to lymph nodes in the head (*7*,*8*). 

**Figure:**
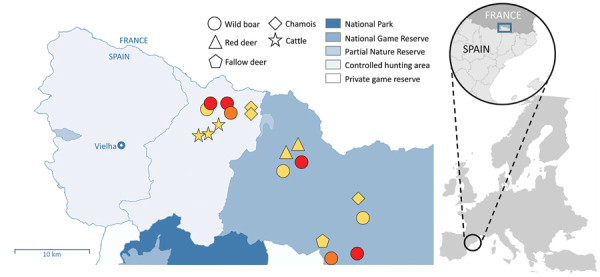
Outbreak area for wild boar tuberculosis (TB) cases, Spain, June 2017–March 2019. Circles show cases of wild boars with TB lesions. Stars indicate the location of cattle herds with positive skin tests (not confirmed at slaughterhouse); triangles, pentagons, and diamonds show locations of *Mycobacterium tuberculosis* complex–seropositive ungulates (no tissue samples were obtained from these animals). Colors indicate etiologic agent identification: red, *M. microti*; orange, *M. tuberculosis* complex (species unidentified); yellow, unidentified. Different hunting areas are indicated. The border between Spain and France and the main village (Vielha) are labelled. Inset maps show location of the study area on the Iberian Peninsula.

To confirm the causative agent for these infections, we extracted DNA from tissue samples (DNAExtract-VK, Vacunek, http://vacunek.com) and performed real-time PCR (TBC-VK, Vacunek), which confirmed MTBC in 6 of 9 suspected cases. DVR spoligotyping at VISAVET Health Surveillance Centre, Universidad Complutense de Madrid, identified the pathogen in 4 of the 6 confirmed MTBC cases as *M. microti* (spoligopattern SB0423; *Mycobacterium bovis* spoligotype database, http://www.mbovis.org). In the other 2 cases, the laboratory was unable to determine the species because of low DNA load from the sample. This result likely was due to the slow in vitro growth rate of *M. microti* in infected animals, which makes it difficult to isolate in routine diagnostic laboratories. 

Spoligopattern SB0423 is included in a phylogenetic cluster with spoligopattern SB0112, also associated with *M. microti*, on the basis of neighbor joining. Both spoligotypes are localized in the eastern French Pyrenees, close to the borders with Spain and Andorra (*3*). Most *M. microti* cases in France, found in cats, dogs, and llamas during 2005–2016 and, since 2015, in wild boars and badgers, have been found within 50 km of the outbreak area in Spain. The 2017 *M. microti* cases described in this report were found closer to the border with France; the remaining 2 cases, detected in 2019, were localized near the southern limit of the outbreak area (Figure). 

In the outbreak area, up to 18 animals in 3 cattle herds showed positive results for a single intradermal tuberculin skin test (Figure). However, none of these animals showed gross lesions in target tissues (i.e., lungs, pulmonary, and retropharyngeal lymph nodes) at a slaughterhouse or positive results to mycobacterial culture and PCR. Similarly, in a recent case in France, a cow reacting to a TB skin test did not show TB-like lesions in respiratory tissues and returned negative results from cultures, but *M. microti* DNA was finally detected in retropharyngeal lymph nodes only by using advanced molecular techniques (*5*). These results indicate that cattle exposed to *M. microti* may induce positive results to diagnostic tests performed in bovine TB eradication campaigns. *M. microti* infection can sometimes cause visible lesions in cattle (*2*), but the fact that *M. microti* are natural knockouts for the virulence-related RD1^mic^ genomic region (*10*) may indicate a lower pathogenicity compared with other MTBC species and account for these negative test results. 

We tested additional wild ungulates in the outbreak area and found that 6 (2 red deer, 1 fallow deer, and 3 chamois) were seropositive for MTBC using a MPB83-specific IgG indirect ELISA test (Figure). Unfortunately, no tissue samples were submitted to examine for lesions or to detect and identify mycobacteria. However, overall positive results for *M. microti* and the absence of other MTBC strains during the 2017–2019 period in the outbreak area suggest a multihost circulation of *M. microti*. Because voles are known maintenance hosts of *M. microti* (*1*), further investigation of wild small rodent populations in the Outbreak area could determine the epidemiology of this outbreak in greater detail. 

These findings, together with previously reported cases nearer the border between France and Spain, indicate a transboundary circulation of *M. microti* across the Pyrenean border that should be taken into account for wildlife TB surveillance. Coordinated action between animal health authorities and laboratories in Spain and France is required, as well as the improvement of livestock management and biosecurity practices. 

## References

[1] Cavanagh R, Begon M, Bennett M, Ergon T, Graham IM, De Haas PE, et al. *Mycobacterium microti* infection (vole tuberculosis) in wild rodent populations. J Clin Microbiol. 2002;40:3281–5. 10.1128/JCM.40.9.3281-3285.2002 12202566 PMC130808

[2] Smith NH, Crawshaw T, Parry J, Birtles RJ. *Mycobacterium microti*: More diverse than previously thought. J Clin Microbiol. 2009;47:2551–9. 10.1128/JCM.00638-09 19535520 PMC2725668

[3] Michelet L, de Cruz K, Karoui C, Hénault S, Boschiroli M. *Mycobacterium microti*, an unrecognized tubercular agent [in French]. Epidémiol Santé Anim. 2017;71:129–38.

[4] Michelet L, de Cruz K, Phalente Y, Karoui C, Hénault S, Beral M, et al. *Mycobacterium microti* infection in dairy goats, France. Emerg Infect Dis. 2016;22:569–70. 10.3201/eid2203.151870 26890061 PMC4766897

[5] Michelet L, De Cruz K. *Mycobacterium microti* infection in a cow in France. Vet Rec. 2017;180:429. 28450491 10.1136/vr.j2041

[6] Panteix G, Gutierrez MC, Boschiroli ML, Rouviere M, Plaidy A, Pressac D, et al. Pulmonary tuberculosis due to *Mycobacterium microti*: a study of six recent cases in France. J Med Microbiol. 2010;59:984–9. 10.1099/jmm.0.019372-0 20488936

[7] Schöning JM, Cerny N, Prohaska S, Wittenbrink MM, Smith NH, Bloemberg G, et al. Surveillance of bovine tuberculosis and risk estimation of a future reservoir formation in wildlife in Switzerland and Liechtenstein. PLoS One. 2013;8:e54253. 10.1371/journal.pone.0054253 23349839 PMC3549981

[8] Boniotti MB, Gaffuri A, Gelmetti D, Tagliabue S, Chiari M, Mangeli A, et al. Detection and molecular characterization of *Mycobacterium microti* isolates in wild boar from northern Italy. J Clin Microbiol. 2014;52:2834–43. 10.1128/JCM.00440-14 24871212 PMC4136184

[9] Chiari M, Ferrari N, Giardiello D, Avisani D, Pacciarini ML, Alborali L, et al. Spatiotemporal and ecological patterns of *Mycobacterium microti* infection in wild boar (*Sus scrofa*). Transbound Emerg Dis. 2016;63:e381–8. 10.1111/tbed.12313 25580561

[10] Brodin P, Eiglmeier K, Marmiesse M, Billault A, Garnier T, Niemann S, et al. Bacterial artificial chromosome-based comparative genomic analysis identifies *Mycobacterium microti* as a natural ESAT-6 deletion mutant. Infect Immun. 2002;70:5568–78. 10.1128/IAI.70.10.5568-5578.2002 12228284 PMC128332

